# Analysis of different innovative formulations of curcumin for improved relative oral bioavailability in human subjects

**DOI:** 10.1007/s00394-016-1376-9

**Published:** 2017-02-16

**Authors:** Martin Purpura, Ryan P. Lowery, Jacob M. Wilson, Haider Mannan, Gerald Münch, Valentina Razmovski-Naumovski

**Affiliations:** 1 Increnovo LLC, 2138 E Lafayette Pl, Milwaukee, WI 53202 USA; 2 Department of Health Sciences and Human Performance, The University of Tampa, Tampa, FL 33606 USA; 3 National Institute of Complementary Medicine, Western Sydney University, Campbelltown, NSW 2560 Australia; 4 South Western Sydney Clinical School, School of Medicine, The University of New South Wales, Sydney, NSW 2052 Australia; 5 Molecular Medicine Research Group, School of Medicine, Western Sydney University, Campbelltown, NSW 2560 Australia; 6 Centre for Health Research, School of Medicine, Western Sydney University, Campbelltown, NSW 2560 Australia

**Keywords:** Curcumin, Cyclodextrin, Bioavailability, Humans, Plasma pharmacokinetics

## Abstract

**Purpose:**

The optimal health benefits of curcumin are limited by its low solubility in water and corresponding poor intestinal absorption. Cyclodextrins (CD) can form inclusion complexes on a molecular basis with lipophilic compounds, thereby improving aqueous solubility, dispersibility, and absorption. In this study, we investigated the bioavailability of a new γ-cyclodextrin curcumin formulation (CW8). This formulation was compared to a standardized unformulated curcumin extract (StdC) and two commercially available formulations with purported increased bioavailability: a curcumin phytosome formulation (CSL) and a formulation of curcumin with essential oils of turmeric extracted from the rhizome (CEO).

**Methods:**

Twelve healthy human volunteers participated in a double-blinded, cross-over study. The plasma concentrations of the individual curcuminoids that are present in turmeric (namely curcumin, demethoxycurcumin, and bisdemethoxycurcumin) were determined at baseline and at various intervals after oral administration over a 12-h period.

**Results:**

CW8 showed the highest plasma concentrations of curcumin, demethoxycurcumin, and total curcuminoids, whereas CSL administration resulted in the highest levels of bisdemethoxycurcumin. CW8 (39-fold) showed significantly increased relative bioavailability of total curcuminoids (AUC_0−12_) in comparison with the unformulated StdC.

**Conclusion:**

The data presented suggest that γ-cyclodextrin curcumin formulation (CW8) significantly improves the absorption of curcuminoids in healthy humans.

## Introduction

*Curcuma longa L*. (Zingiberaceae), known as turmeric, has been used in the traditional medicine in China and India for centuries. Turmeric consists of natural bioactive hydrophobic polyphenols called curcuminoids of which curcumin is the main component derived from the rhizome of the herb. With its extensive pharmacological activities, including antioxidant, anti-cancer, antimicrobial, anti-inflammatory, and anti-diabetic properties [1–3], a variety of studies have investigated the mode of action of curcumin in signal transduction pathways linked to inflammation. For example, curcumin has been shown to inhibit IL-6-induced STAT3 phosphorylation and consequent STAT3 nuclear translocation in multiple types of myeloma cell lines [4]. In addition, cell culture studies have shown that curcumin prevents TNF-α-induced IL-1 and IL-6 production by interfering with the nuclear factor kappa-light-chain enhancer of activated B cells (NF-κB) and mitogen-activated protein kinases (MAPK) pathways [5–7]. In microglia and macrophage cell lines, curcumin also has shown to have an inhibitory effect on cyclooxygenase-2 (COX-2) and inducible nitric oxide synthase (iNOS) expression, leading to decreased levels of prostaglandin E_2_ (PGE2) and nitric oxide (NO) [7–9]. Furthermore, curcumin decreases TNF-α, IL-1, -2, -8, and -12 production in phorbol myristate acetate (PMA) or lipopolysaccharide (LPS) stimulated monocytes and alveolar macrophages in a concentration- and time-dependent manner, depicting its broad cytokine-suppressive anti-inflammatory action [10].

**Fig. 1:**
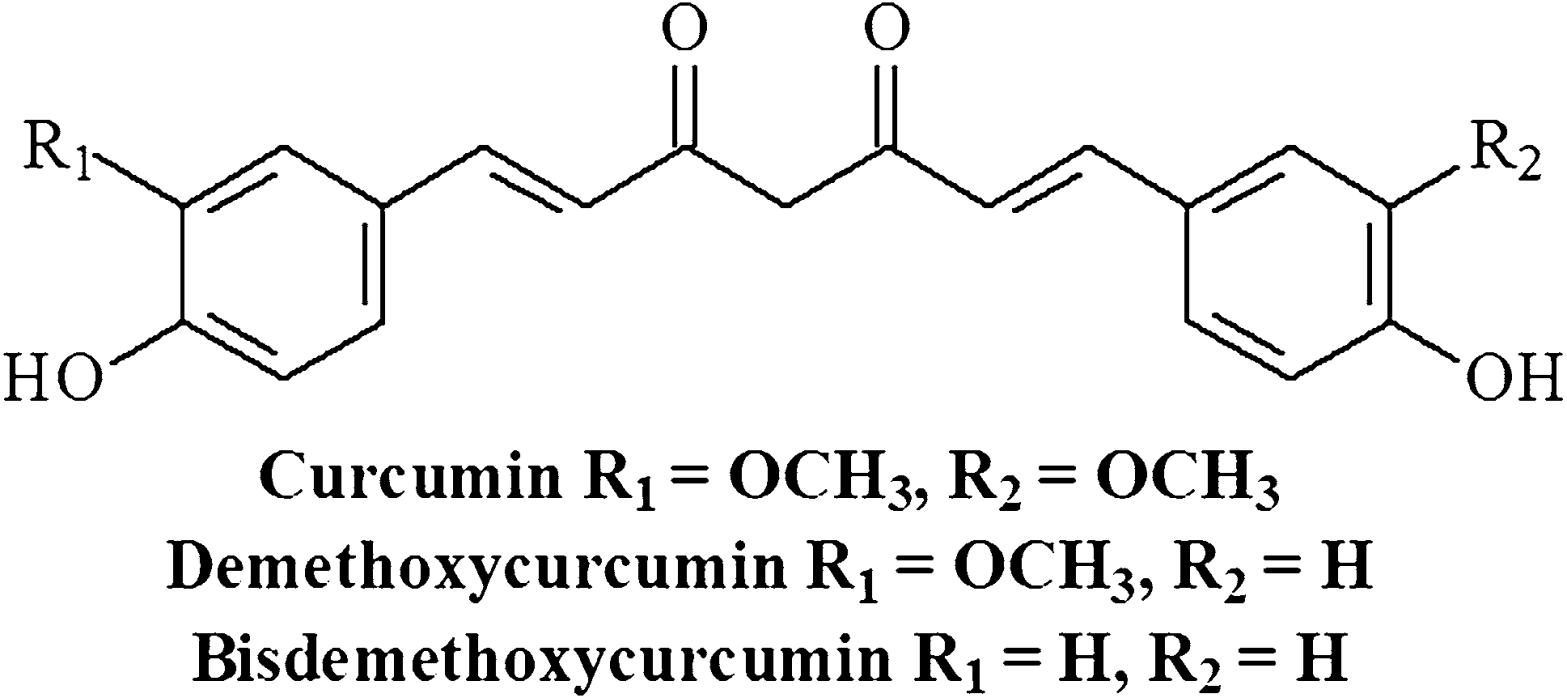
Chemical structures of the curcuminoids. The main curcuminoids isolated from the curcuma longa rhizome are curcumin, demethoxycurcumin (one O–CH3 group replaced by H), and bisdemetoxycurcumin (two O–CH_3_ groups replaced by H)

While early human clinical trials showed beneficial effects for cancer [11, 12], arthritis [13], immune deficiencies [14], and cardiovascular health [15], its potential seems to be limited by poor absorption [16]. Curcumin is practically insoluble in water resulting in insufficient absorption from the gut, and pharmacokinetic studies showed a fast metabolism and quick systemic elimination [17]. The maximal total curcumin plasma concentration in humans reported in the literature was 3228.0 ± 1408.2 ng/ml when 410 mg curcumin was given as liquid micelles [18]. The majority of orally ingested curcumin is excreted through the faeces in a non-metabolised form [19]. Absorbed curcumin and its metabolites are rapidly converted into water-soluble metabolites, glucuronides, and sulfates [20, 21]. Through an NADPH-dependent mechanism and reduction, curcumin is converted into dihydrocurcumin and tetrahydrocurcumin (Fig. 1) [22, 23].

**Fig. 2:**
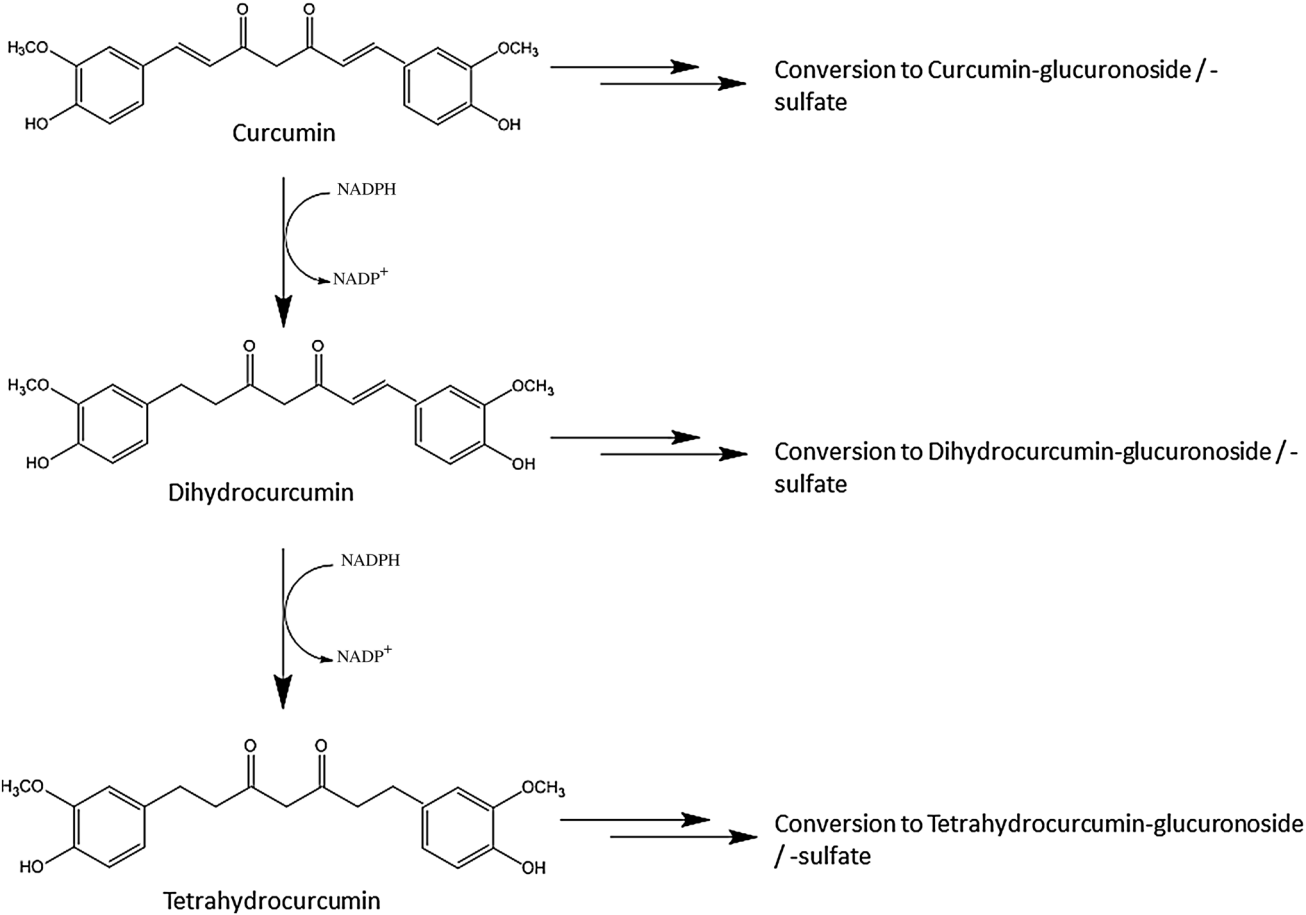
Metabolic pathway of orally ingested curcumin. Curcumin and its reduced metabolites dihydrocurcumin and tetrahydrocurcumin are conjugated with glucuronide and/or sulfate, resulting in curcumin glucuronoside, dihydocurcumin glucuronoside, tetrahydrocurcumin glucuronoside, or corresponding monosulfate and mixed sulfate/glucuronosides

The gut microbiota plays an important role in curcumin metabolimn and biotranformation, as the microbiota is capable of transforming curcumin formulations which contain approximately 77% curcumin, 17% demethoxycurcumin, and 6% bisdemethoxycurcumin (Fig. 2) into a range of catabolites [16]. For example, Tan et al. investigated the colonic metabolism of three curcuminoids [80.1% curcumin, 15.6%, demethoxycurcumin (DMC), and 2.6% bisdemethoxycurcumin (Bis-DMC)] in an in vitro model containing human faecal starters. Up to 24% of curcumin, 61% of demethoxycurcumin (DMC), and 87% of bisdemethoxycurcumin (Bis-DMC) were degraded by the human faecal microbiota after 24 h of fermentation in vitro. Three main metabolites, namely, tetrahydrocurcumin (THC), dihydroferulic acid (DFA), and 1-(4-Hydroxy-3-methoxyphenyl)-2-propanol, were detected in the fermentation cultures [24].

**Fig. 3:**
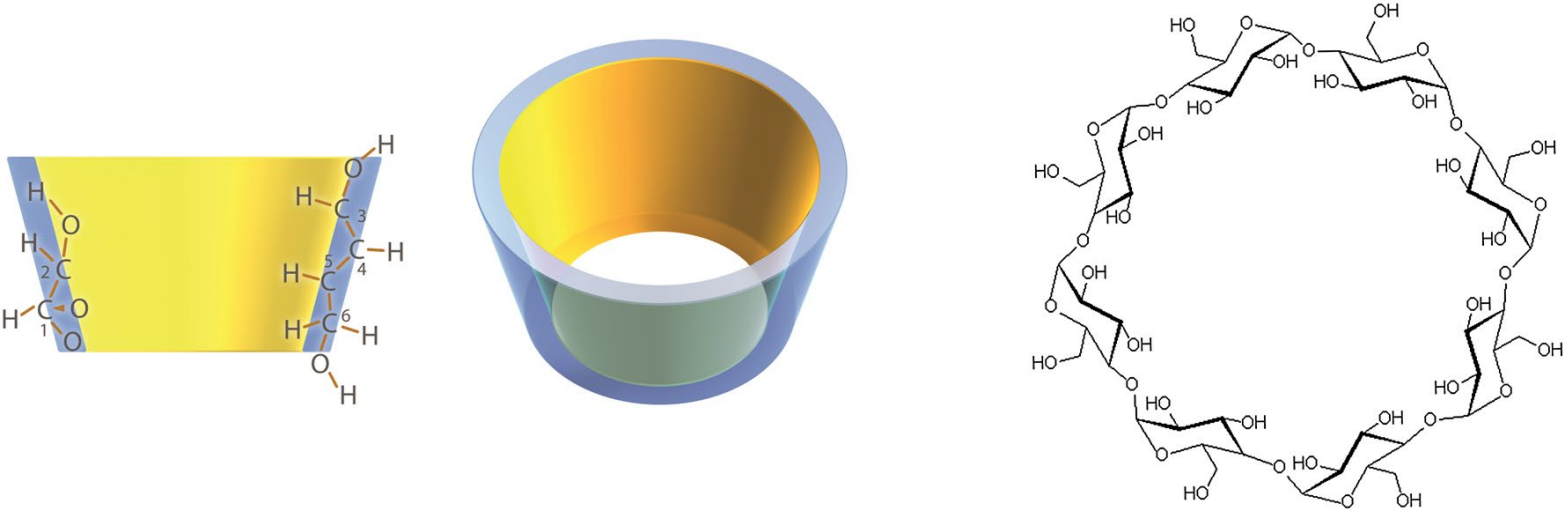
Structure of cyclodextrins. Cyclodextrins are cyclic oligosaccharides consisting of (α-1,4)-linked α-D-glucopyranose units. The corresponding structure of the hydrophilic glucose building blocks face outwards and results in a lipophilic cavity on the inside. The size and shape of the cavity allow a lipophilic molecule to reside as a “guest”. The cohesion between the cyclodextrin and the guest molecules is produced by relatively weak van der Waals forces, so that the guest molecule can be liberated again under suitable conditions

Cyclodextrins have been widely used in pharmaceutical and nutritional formulations to form an inclusion complex on a molecular basis with lipophilic compounds for the improvement of their aqueous dispersibility and corresponding bioavailability [25]. α-, β-, and γ-Cyclodextrin are a family of cyclic oligosaccharides consisting of non-reducing chiral glucose building blocks linked into a ring. The corresponding structure of the hydrophilic glucose building blocks face outwards and results in a lipophilic cavity on the inside (Fig. 3). The size and shape of the cavity allow a lipophilic molecule to reside as a “guest”. The cohesion between the cyclodextrin and the guest molecules is produced by relatively weak van der Waals forces, so that the guest molecule can be liberated again under suitable conditions. The weak van der Waals forces in such inclusion complexes leave the two counterpart molecules unchanged and in equilibrium. About 30 different pharmaceutical products containing cyclodextrins are now on the market worldwide, and numerous food products, cosmetics, and other commercial products contain cyclodextrins. In these products, cyclodextrins are mainly used solubilizing agents to increase water solubility of lipophilic compounds [26].

In contrast to α- and β-cyclodextrin, γ-cyclodextrin is completely digested by salivary and pancreatic amylase. In animals, the administration of tocotrienol-γ-cyclodextrin complex resulted in higher plasma and tissue tocotrienol concentrations by enhancing intestinal absorption [27]. After a single dose of a capsule containing the inclusion complex of coenzyme Q10 with γ-cyclodextrin, the coenzyme Q10 plasma levels were significantly elevated [28]. These findings suggest that the complexation of lipophilic curcumin with γ-cyclodextrin may improve its bioavailability.

Therefore, the purpose of this study was to evaluate the plasma levels of curcuminoids (curcumin, demethoxycurcumin, and bisdemethoxycurcumin) of an acute oral administration of a novel curcumin-γ-cyclodextrin complex containing curcumin (CW8) in comparison with standard unformulated curcumin (StdC). In addition, a curcumin phytosome formulation consisting of Curcumin: Soy Lecithin: Microcrystalline Cellulose in a ratio of 1:2:2 (CSL) and a formulation consisting of curcuminoids and essential oils of turmeric rhizome (CEO) were also included in this study.

## Materials and methods

### Subjects

Out of 15 subjects recruited for this study, 12 subjects completed the study (11 male, 1 female; age 23.0 ± 2.4 years; height 182.9 ± 6.1 cm; weight 86.2 ± 4.2 kg, 1 African American and 11 Caucasians). One volunteer did not start the study and another volunteer dropped out of the study due to personal reasons. During blood withdrawal, another volunteer was feeling faint and, therefore, was advised not to proceed with the study. Volunteers participating in the study needed to meet the following inclusion parameters: 20–35 years of age have not been consuming any curcumin-containing supplements (curcumin, turmeric, and curry) or foods (curcumin, turmeric, and curry for 2 weeks prior to testing; no history of any of the following: hyperacidity, gastric/duodenal ulcers, gastrointestinal problems, and gallbladder problems; no use of any blood thinners/anti-thrombotic agents or NSAIDs; no prior use of blood sugar-lowering agents, H2 blockers, or proton pump inhibitors; non-hyperglycaemic, non-haemophiliac, and non-diabetic; and no known allergies to soy. The University of Tampa Institutional Review Board approved the protocol (IRB, 07/02/2013, Ref: 13-07). Before each testing, all subjects underwent screening and signed informed written consent to guarantee eligibility and voluntary willingness to take part.

### Study materials

Product names have been omitted due to the absence of consent for disclosure. The total mass of each of the preparations was matched by using inert filler material (microcrystalline cellulose). All volunteers were supplemented with visually identical six hard gel capsules of each of the study materials per setting, resulting in either 376 mg of total curcuminoids for CW8, CSL and CEO and 1,800 mg of total curcuminoids for StdC in accordance with the study dosage established by the Cuomo et al. [19]. Prior to the study, capsules of each product were analysed and the actual amount of the curcuminoids per serving was calculated as mean values (Table 1).

**Table 1:** Analytical results of study materials

	StdC (mg)	CSL (mg)	CEO (mg)	CW8 (mg)
Curcumin	1774.2	354.0	355.2	348.0
Demethoxycurcumin	162.0	26.4	35.4	21.6
Bisdemethoxycurcumin	9.0	1.2	1.8	2.4
Total Curcumioids	1945.2	382.2	392.4	371.4

### Study procedure

All 12 subjects completed the four separate trials of the four formulations, with nine blood samples drawn from each in 1 day, in a randomized, double-blinded order separated by a 7-day wash-out period between each formulation. The curcumin formulations were blinded through a special code, so that the investigators, as well as the volunteers, did not know which formulation was consumed during each session.

**Fig. 4:**
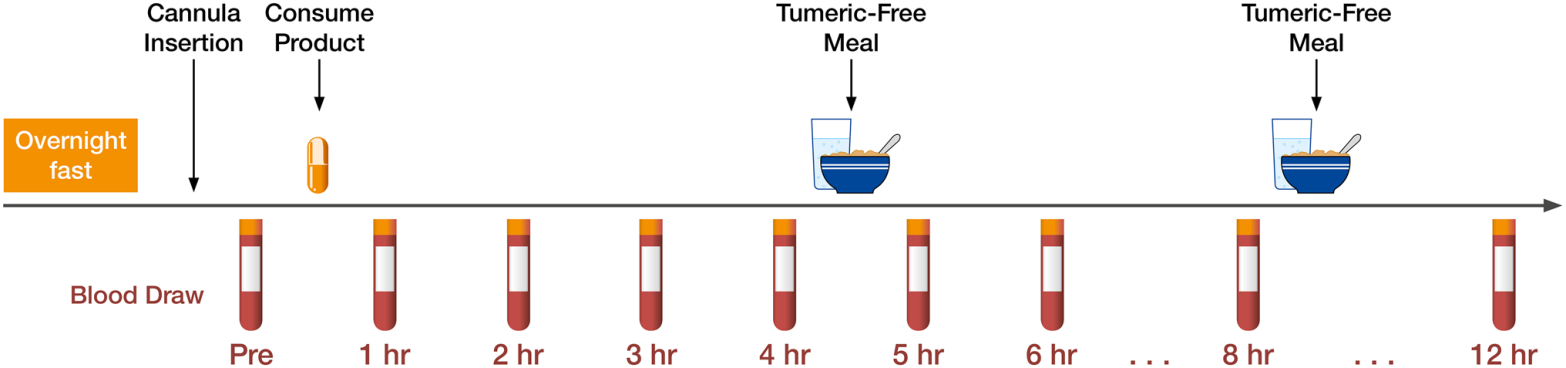
Schematic representation of the study protocol. Each volunteer reported to the laboratory in the morning between 6:00 and 10:00 h following a 10-h overnight fast (except for water). A catheter was introduced into a forearm vein by a qualified phlebotomist. After equilibration, a baseline blood sample (pre) was collected and one of four treatment dosages of curcumin was consumed with water. Blood samples were then drawn at 1, 2, 3, 4, 5, 6, 8, and 12-h intervals following product consumption. After the 4- and 8-h blood samples had been drawn, a turmeric-free standardized meal was provided

Before each trial, the subject reported to the laboratory in the morning following a 10-h overnight fast (except for water). Blood was drawn by introducing a catheter into the forearm vein by a qualified phlebotomist. First, the baseline blood sample was obtained, followed by one of four treatments with the defined four curcumin preparations which were consumed with water. Further blood samples were then drawn at the timepoints of 1, 2, 3, 4, 5, 6, 8, and 12 h following product supplementation (Fig. 4). These timepoints were selected as past studies have shown that the majority of digestion and absorption are practically complete within this timeframe. Each time after the 4- and 8-h blood sample draw, a curcumin-free standardized meal was delivered. During the first mealtime, 40 g chocolate whey protein isolate and 80 g instant oatmeal dissolved in 30 mL of water plus 473 mL of water to drink were served. During the second mealtime, 230 g turkey breast, 2 slices of whole wheat bread, 15 g light miracle whip, 170 g of fat free Greek yogurt, and 473 mL of water to drink were served. All subjects remained in the laboratory the entire experiment to ensure full compliance. .

### Sample collection

At each blood withdrawal timepoint, 6 mL of blood were drawn off the catheter into vacutainer tubes, followed by centrifugation of the blood tubes at 2000×*g* for 10 min and the plasma was aliquoted into Eppendorf tubes for storage until analysis. To avoid degradation during the storage, the blood plasma samples were stored in a −80 °C freezer until analysis.

### Sample preparation

The plasma samples were prepared according to Cuomo et al. [29]. A 0.2 mL aliquot of plasma was transferred to a clean microcentrifuge tube and spiked with 100 μL of a solution containing 1000 U of β-glucuronidase/sulfatase (EC 3.2.1.31) from *Helix pomatia* (Sigma, St. Louis, MO) in 0.1 M phosphate buffer (pH 6.86) and 50 μL of methanol to liberate free curcumin [30], as a substantial amount of curcumin is glucuronidated or sulfated [23]. For enzymatic hydrolysation of the phase-2 conjugates of curcuminoids, the resultant mixture was thoroughly vortexed and incubated at 37 °C for 1 h. In a subsequent incubation, curcuminoids were extracted with 1 mL of ethyl acetate, and the mixture was then vortexed for 1 min, followed by sonication in a water bath for 15 min. After 6 min of centrifugation at 15,000*g*, the resulting upper organic layer was transferred to a 2 mL microcentrifuge tube and evaporated at 30 °C under negative pressure in a centrifugal concentrator to remove residual solvent. This extraction procedure was repeated for a total of two extractions. After treating the dried extract with 100 μL of methanol, 10 μL were injected into the HPLC-MS/MS. “Salbutamol” (ISTD) was used as an internal standard to ensure data accuracy. All standard curcuminoids for the quantification were purchased from Sigma Aldrich, USA.

### Chromatographic analysis of the curcuminoids

Blood plasma samples were analysed by tandem mass spectrometry detection (HPLC/MS/MS) to determine curcumin, demethoxycurcumin, and bisdemethoxycurcumin levels as published previously [29, 31, 32]. Briefly, the HPLC-MS-MS consisted of an Agilent 1290 HPLC system with an Agilent 6460 tandem mass spectrometer with ESI source in positive mode. Chromatographic separation was achieved by a Kinetex XB-C18 100 Å column (2.1 × 50 mm, 2.6 micron) attached to a security guard ultra, C18, 2.1 mm pre-column. The column chamber temperature was set to 50 °C. The mass spectrometer was run in the multiple reaction monitoring (MRM) mode, and the transitions monitored were m/z 369.1 → 285.1 for curcumin, 339.1 → 255.1 for demethoxycurcumin, and 309.1 → 225.0 for bisdemethoxycurcumin. Concentrated stock solutions of curcumin, demethoxycurcumin, and bisdemethoxycurcumin were prepared by dissolving 5.0 mg of each compound in 200 mL of methanol to give 25 μg/mL stock solutions. Calibration standards were prepared daily by spiking 1 mL of blank plasma with the appropriate working solution resulting in concentrations of 0.5, 50, 100, 200, and 500 ng of curcumin, its derivaties and salbutamol per ml plasma as described previously [32]. A six-point calibration curve was created by plotting the peak area ratio (y) of curcumin to the internal standard salbutamol vs. the curcumin concentration. The calibration curves were linear in human plasma with curves (*r* = .99) for curcumin. Similar results were obtained for demethoxycurcumin and bisdemethoxycurcumin. The analysis was carried out in a water/methanol gradient, and the flow rate was 0.25 mL/min and the mobile phase was mixed from two components: A—water containing 0.1% formic acid; B—methanol containing 0.1% formic acid. Gradient conditions were: 70% B at 0 and 3 min, increasing to 98% B at 5 and 6 min, before returning back to 70% B at 7.5 min. Salbutamol (50 µg/mL) was used as an internal standard as described previously [32]. Blank human serum was pooled together and stored at −20 °C prior to use for the preparation of calibration standards and quality control samples.

### Pharmacokinetic analysis

Pharmacokinetic data following the oral administration of the curcumin formulations were calculated by Graphpad Prism 5 and PKSolver using non-compartmental analysis [33]. C_max_ was the maximum observed plasma concentration directly from the mean plasma concentration time profile (median are also presented in and the area under the plasma concentration time curve (AUC) was calculated by the definite integral from 0 to 12 h of the mean plasma concentration time curves using the additive method. StdC was given to the participants at approximately five times the dose; therefore, normalized values for StdC (where normalized C and AUC_0−12_ values were divided by the content of each curcuminoid for each preparation divided by the curcumin content of the larger dose of StdC) are also presented in Table 1 and used for the statistical comparisons. Calculation of *t*_½_ could not take place for all curcumin preparations as a number of the formulations did not decline in concentration over the 12-h time period. Both programs yielded identical values for AUC_0−12_ and C_max_ (additive method).

### Statistical analysis

**Fig. 5:**
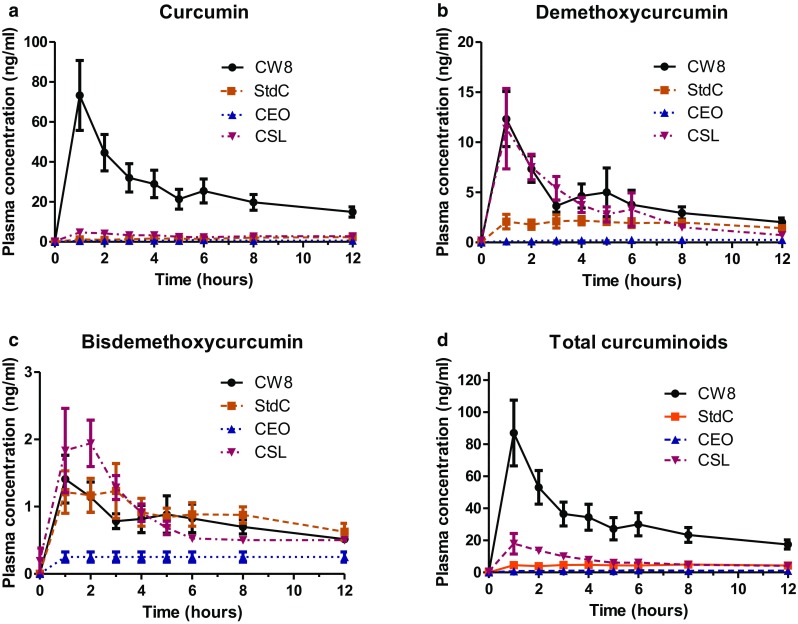
Plasma concentration time curves for curcumin, demethoxycurcumin, bisdemethoxycurcumin, and total curcuminoids for the four different curcumin formulations. Pharmacokinetic data of the individual and combined total curcuminoids for the four formulations were each plotted on a plasma concentration vs. time curve. From these data, the area under the plasma concentration time curve (AUC), *C*_max_, *t*_max,_ and relative absorption was calculated for each curcuminoid and the combined curcuminoids. Concentrations (means ± SEM) are expressed in ng/mL and refer to enzymatically hydrolyzed plasma samples

The data were expressed as mean ± SEM and pharmacokinetics analysis performed using Pk solver and Graphpad Prism 5 (Fig. 5). A second analysis is also presented (Table 2) using the median (IQR), as the outcome was non-normal in each occasion and Friedman’s test was performed to examine whether the outcome varied significantly by the four types of curcumin formulations examined in this study. For the median, Friedman’s test is a type of non-parametric-repeated-measures one-way ANOVA. If it was significant, the parameters were compared in pairs by the non-parametric sign test adjusted for multiple comparisons using the Bonferroni method. Signed rank test or paired *t* test was not performed as the outcome for each comparison was neither symmetrical nor normal Out of the six all possible comparisons between any two types of curcumin concentrations, the comparison between CEO and CSL was not of interest. This produced five comparisons—between StdC and CW8, CEO, and CSL and between CW8 and CEO, CSL. In total, four-repeated-measures non-parametric ANOVA models were fitted for Cmax (one each for curcumin, demethoxycurcumin, bisdemethoxycurcumin, and total curcuminoids). Similarly, four-repeated-measures non-parametric ANOVA models were fitted for AUC. The adjusted *p* values of less than 0.05 were considered statistically significant for each pairwise comparison.

## Results

To improve the bioavailability of curcumin, many different approaches, including the design and development of nanoparticles, self-assemblies, nanogels, liposomes, and complex fabrication, have been developed for sustained and efficient curcumin delivery [32].

In our study, we have compared four different commercially available curcumin formulations and analysed their pharmacokinetic profile in 12 subjects in a randomized, double-blind, cross-over study over a 12-h time period.

The subjects consumed either 376 mg of total curcuminoids in the form of CW8, CEO, and CSL or 1,800 mg of the corresponding non-formulated StdC. All four treatments were well tolerated, and no adverse events were reported.

Curcumin, demethoxycurcumin, and bisdemethoxycurcumin plasma levels were measured by HPLC-MS/MS analysis after Helix pomatia glucuronidase/sulfatase treatment to liberate the parent compounds from sulfate and glucoronidate conjugates.

For each formulation, the pharmacokinetic data of the individual curcuminoids were plotted on a plasma concentration vs. time curve (Fig. 5). The area under the plasma concentration time curve (AUC), *C*_max_, *t*_max,_ and relative bioavailability (*F*) were calculated for each curcuminoid in the four formulations (Table 2). The relative bioavailability was calculated by dividing the measured value of test product (CSL, CEO, or CW8) by the measured value of reference product (StdC) multiplied by the dosage of the reference product divided by dosage of the test product.

CW8 showed the highest mean plasma concentrations of curcumin and total curcuminoids (73.2 ± 17.5 and 87.0 ± 20.5 ng/mL, respectively), whereas CSL administration resulted in the highest mean plasma levels of bisdemethoxycurcumin (1.9 ± 0.3 ng/mL). For demethoxycurcumin, CW8 and CSL yielded the highest mean plasma concentrations (12.4 ± 2.7 and 5.0 ± 0.7 ng/mL, respectively). CW8 and CSL reached C_max_ at 1 h after administration, whereas the other formulations showed a delayed uptake (Fig. 5; Table 2).

**Table 2:** Pharmacokinetic parameters of curcuminoid concentrations: area under the plasma concentration time-curve (AUC_0–12h_), *C*_max_, and relative absorption for each treatment

Curcuminoid	Formulation	AUC_0-12_ (ng/mL h) (means ± SEM)	*C*_max_ (ng/mL) (means ± SEM)	AUC_0-12_ (ng/mL h) (median, IQR)	*C*_max_ (ng/mL) (median, IQR)	*t*_max_ (h)	Relative absorption (fold)
Curcumin	StdC (5x dose)	19.7 ± 2.6	2.3 ± 0.4	19.7 (2.6)	0.3 (0.3)	12	1.0
	StdC (normalized)	3.9 ± 0.5	0.5 ± 0.1	3.5 (2.2)	0.0^f^ (0.5)	12	1.0
	CEO	6.7 ± 0.5	0.9 ± 0.3	5.8^f^ (2.2)	5.2^c^ (18.9)	6	1.7
	CSL	35.1 ± 4.5	4.7 ± 1.8	34.2^f^ (25.1)	10.4^c ^(13.6)	1	9.0
	CW8	327.7 ± 58.1	73.2 ± 17.5	293.4^c^ (205.8)	1.2 (0.4)	1	85.0^c^
Demethoxy-curcumin	StdC (5x dose)	21.8 ± 3.1	2.2 ± 0.4	21.8 (3.1)	0.1 (0.2)	4	1.0
	StdC (normalized)	3.8 ± 0.5	0.4 ± 0.1	3.7 (2.9)	0.3^e^ (0.5)	4	1.0
	CEO	2.5 ± 0.8	0.3 ± 0.1	1.3^f^ (5.5)	2.0^c^ (2.3)	6	0.5
	CSL	41.8 ± 7.5	5.0 ± 0.7	37.2^c^ (25.4)	1.1^c^ (1.8)	6	11.9^c^
	CW8	51.5 ± 9.0	12.4 ± 2.7	43.6^c^ (43.1)	4.7 (0.8)	1	17.9^c^
Bisdemethoxy-curcumin	StdC (5x dose)	10.6 ± 1.4	1.2 ± 0.4	10.6 (1.4)	0.8 (0.7)	1	1.0
	StdC (normalized)	2.1 ± 0.3	0.2 ± 0.1	2.0 (1.8)	1.0^f^ (0.4)	1	1.0
	CEO	2.9 ± 0.8	0.3 ± 0.1	3.4^d^ (5.8)	8.1^c,f^ (27.3)	n/a	1.4
	CSL	10.0 ± 1.0	1.9 ± 0.3	9.4^a^ (4.4)	67.8^c^ (93.2)	2	7.1^c^
	CW8	9.4 ± 1.3	1.4 ± 0.3	8.0^c^ (4.6)	0.3 (0.3)	1	3.3^c^
Total curcuminoids	StdC (5x dose)	52.1 ± 6.4	4.7 ± 0.8	52.1 (6.4)	0.0^f^ (0.5)	4	1.0
	StdC (normalized)	10.4 ± 1.3	0.9 ± 0.1	10.6 (6.5)	5.2^c^ (18.9)	4	1.0
	CEO	12.1 ± 1.4	1.1 ± 0.1	11.4^f^ (6.8)	10.4^c^ (13.6)	4	1.1
	CSL	86.9 ± 12.1	18.0 ± 6.4	88.1^f^ (45.9)	1.2 (0.4)	1	8.5
	CW8	388.6 ± 66.8	87.0 ± 20.5	343.7^c^ (293.9)	0.1 (0.2)	1	39.1^c^

Data are expressed as mean ± standard errors of the mean or as median (IQR). Significances were calculated based on the medians. *P* values less than 0.05 (a), 0.01 (b) and 0.001 (c) were considered statistically significant (based on pairwise comparisons using signed test with Bonferroni adjustments) in comparison to the Normalized StdC values. *P* values less than 0.05 (d), 0.01 (e) and 0.001 (f) were considered statistically significant (based on pairwise comparisons using signed test with Bonferroni adjustments) in comparison to CW8 values (note that the dose of StdC was approximately 5x that of the three other curcumin formulations, and therefore a normalized value was used for the statistical comparisons)

## Discussion

While most of the formulations are targeting drug delivery, only a few food-grade preparations have been studied [29, 34, 35]. Many different strategies to increase the potential bioavailability of curcumin have been explored in the recent past, including the design and development of nanoparticles, self-assemblies, nanogels, liposomes, and micelles, have been developed for sustained and efficient curcumin delivery [36].

One approach to increase curcumin absorption involves more components of the raw turmeric root to be included in the preparation. For example, the combination of curcuminoids and essential oils of turmeric rhizome (CEO) have been shown to increase the absorption of curcumin by 6.9-fold [34]. The inclusion of curcumin in a lipophilic matrix (Phytosomes, Curcumin:Soy Lecithin:Microcrystalline Cellulose 1:2:2, CSL) has been shown to increase the relative human bioavailability of curcumin by 19.2-fold for curcumin alone [29]. A formulation of curcumin with a combination of hydrophilic carrier, cellulosic derivatives, and natural antioxidants (Curcuwin, OmniActive) has aslo be shown to significantly increased curcuminoid bioavailability compared to unformulated standard curcumin [31].

In this study, four different curcumin preparations were tested side by side with in the same human subjects. CW8 showed the highest plasma concentrations of curcumin, demethoxycurcumin, and total curcuminoids, whereas CSL administration resulted in the highest levels of bisdemethoxycurcumin. CW8 (39.1-fold) showed significantly increased relative bioavailability of total curcuminoids (AUC_0−12_) in comparison with unformulated StdC.

Cuomo et al. showed that the lecithin in CSL increased the uptake of the demethoxylated forms of curcumin into the blood plasma [29]. In the standard curcumin formulations, the curcumin content is four times higher than the amount of demethoxycurcumin; nevertheless, the formulation with lecithin (CSL) results in demethoxycurcumin being the major plasma curcuminoid, and not curcumin. This was emulated in our study for CSL, whereas curcumin was the major plasma curcuminoid for CW8.

However, the pharmacokinetic parameters for CSL cannot be compared to those in the study by Cuomo et al. as both studies show significant differences in study design [29]. The first major difference was the duration of blood sampling. Cuomo et al. drew blood samples over a 24-h period of time, compared to 12 h in this study. The second difference was the nutritional status. The subjects in this study were fasted, while Cuomo et al. gave a high fat meal with the curcumin supplementation which has shown to slow the mean transit time (MTT) in the gastrointestinal tract and, therefore, improve the absorption of fat soluble ingredients. Regarding the analytical method, Cuomo et al. did not use an internal standard. Therefore, in this study, an internal standard, “Salbutamol” (ISTD), was used to improve accuracy and reliability of the data outcome as previously described by Lao et al. 2006 in an absorption study in rats [37]. Due to the differences in design, absolute values between the two studies cannot be compared.

Another curcumin absorption study conducted by Antony et al. showed the effects of a formulation of curcumin with essential oils of turmeric extracted from the rhizome (CEO) and a curcumin–lecithin–piperine over a curcumin control in 11 healthy volunteers in a cross-over design with a 3-week wash-out period [34]. For the analytical method, an internal standard was not applied, resulting in determination of curcumin alone in the blood for up to 8 h after administration. As a result, the formulation showed a 6.9-fold increased absorption over control, whereas in this study, a much lower increase with approximately 30% relative absorption of CEO was demonstrated.

In a dose escalation study conducted by Lao et al., the safety and appearance of curcumin were determined in the blood of a single dose of StdC, the same material used as the control in this study [37]. Twenty-four healthy subjects (*n* = 24) consumed increasing single doses of 500, 1000, 2000, 4000, 6000, 8000, 10,000, and 12,000 mg of StdC. Surprisingly, no curcumin was detected in serum at up to 8 g of StdC. Only at a dose level of 10,000 and 12,000 mg in two volunteers resulted in low levels of curcumin, whereas no curcumin could be detected in the remaining subjects at the 10,000- or 12,000-mg dose levels [37]. A further highly bioavailable curcumin preparation is Longvida, a patented technology using Solid Lipid Curcumin Particle (SLCP™) Technology [38]. Novasol curcumin is incorporated into biomimetic micelle with a diameter of approximately 30 nm with purported bitter bioavailability as liposomal preparations [18].

The absolute values of other studies cannot be compared with the results of this study due to variances in subjects, analytical method, study design, and administration of the product.

This present study is one of the few studies in which four different curcumin preparations were compared in the same cohort of subjects, and where the different curcuminoids in the curcumin formulation (curcumin, bisdemethoxycurcumin, and demethoxycurcumin) were analysed and compared, aided by the use of an internal standard.
